# Blockade of the lncRNA-DOT1L-LAMP5 axis enhances autophagy and promotes degradation of MLL fusion proteins

**DOI:** 10.1186/s40164-024-00488-5

**Published:** 2024-02-19

**Authors:** Tian-Qi Chen, Heng-Jing Huang, Shun-Xin Zhu, Xiao-Tong Chen, Ke-Jia Pu, Dan Wang, Yan An, Jun-Yi Lian, Yu-Meng Sun, Yue-Qin Chen, Wen-Tao Wang

**Affiliations:** 1 MOE Key Laboratory of Gene Function and Regulation, Guangdong Province Key Laboratory of Pharmaceutical Functional Genes, State Key Laboratory of Biocontrol, School of Life Sciences, Sun Yat-sen University, Guangzhou, 510275 China; 2 State Key Laboratory of Oncology in South China, Sun Yat-sen University Cancer Center, Guangdong, Guangzhou 510060 China; 3 School of Life Sciences, Sun Yat-sen University, Guangzhou, 510275 P. R. China

**Keywords:** lncRNA, LAMP5-AS1, DOT1L, LAMP5, Autophagy, *MLL* leukemia

## Abstract

**Background:**

Mixed-lineage leukemia (*MLL*) fusion gene caused by chromosomal rearrangement is a dominant oncogenic driver in leukemia. Due to having diverse *MLL* rearrangements and complex characteristics, *MLL* leukemia treated by currently available strategies is frequently associated with a poor outcome. Therefore, there is an urgent need to identify novel therapeutic targets for hematological malignancies with *MLL* rearrangements.

**Methods:**

qRT-PCR, western blot, and spearman correction analysis were used to validate the regulation of LAMP5-AS1 on LAMP5 expression. In vitro and in vivo experiments were conducted to assess the functional relevance of LAMP5-AS1 in *MLL* leukemia cell survival. We utilized chromatin isolation by RNA purification (ChIRP) assay, RNA pull-down assay, chromatin immunoprecipitation (ChIP), RNA fluorescence in situ hybridization (FISH), and immunofluorescence to elucidate the relationship among LAMP5-AS1, DOT1L, and the *LAMP5* locus. Autophagy regulation by LAMP5-AS1 was evaluated through LC3B puncta, autolysosome observation via transmission electron microscopy (TEM), and mRFP-GFP-LC3 puncta in autophagic flux.

**Results:**

The study shows the crucial role of LAMP5-AS1 in promoting *MLL* leukemia cell survival. LAMP5-AS1 acts as a novel autophagic suppressor, safeguarding MLL fusion proteins from autophagic degradation. Knocking down LAMP5-AS1 significantly induced apoptosis in MLL leukemia cell lines and primary cells and extended the survival of mice in vivo. Mechanistically, LAMP5-AS1 recruits the H3K79 histone methyltransferase DOT1L to *LAMP5* locus, directly activating LAMP5 expression. Importantly, blockade of LAMP5-AS1-LAMP5 axis can represses MLL fusion proteins by enhancing their degradation.

**Conclusions:**

The findings underscore the significance of LAMP5-AS1 in MLL leukemia progression through the regulation of the autophagy pathway. Additionally, this study unveils the novel lncRNA-DOT1L-LAMP5 axis as promising therapeutic targets for degrading MLL fusion proteins.

**Supplementary Information:**

The online version contains supplementary material available at 10.1186/s40164-024-00488-5.

## Background

The juxtaposition of Mixed-lineage leukemia (*MLL*) genes with various partner genes results in the generation of MLL fusion proteins, which are known to induce aggressive forms of leukemia [1–4]. *MLL* leukemia represents an extremely aggressive subtype of acute leukemia, accounting for up to 70% of infant leukemia cases and 7–10% of adult cases [1, 2]. Due to the diverse chromosomal rearrangements that involve more than 100 partner genes with the *MLL* gene, *MLL* leukemia exhibits complex biological and clinical characteristics [1, 3–7]. This complexity represents an independent dismal prognostic factor, limiting treatment options and leading to poor outcomes [1, 6, 8–10]. Therefore, there is an urgent need to identify novel and effective strategies for the treatment of *MLL* leukemia.

Recent advances highlighted that long noncoding RNAs(lncRNAs)plays key regulatory roles in various cellular and molecular mechanisms in cancer progression, through their modulation of protein expression, aggregation, phase separations, enzyme activation and the chromatin regulatory pathway [11–17]. Furthermore, lncRNAs often exhibit tissue-specific expression and are specific to particular cancer types [11, 14–16, 18]. For example, the upregulation of HOTAIR can activate FGFR and Wnt/β-catenin signaling pathways to promote epithelial–mesenchymal transition (EMT) and inhibit the Akt/JNK signaling pathway, facilitating invasiveness and metastasis in breast cancer [18, 19]. Metastasis-associated lung adenocarcinoma transcript-1 (MALAT1) promotes colorectal cancer cell proliferation, migration, metastasis, and angiogenesis by targeting several genes and signaling pathways, including WNT/β-catenin, Snail, PI3K/AKT/mTOR, PERK/ATF4 [20, 21]. The manipulation of MALAT1-mediated pathways offers a critical therapeutic strategy for colorectal cancer [20]. lncRNA HOTTIP is highly expressed in acute myeloid leukemia (AML) to mediate HOXA topologically associated domain (TAD) formation and mediated R-loop formation to drive oncogene transcription [22, 23]. These studies collectively reveal that lncRNA stands as a fascinating molecule in cancer biology. Comprehensive understand of the multifaceted functions and diverse implications of lncRNA in carcinogenesis will make it a promising and innovative approach for malignancy treatment.

In our previous research, we identified a specific lncRNA called LAMP5-AS1 that is overexpressed in *MLL* leukemia patients [24]. We have shown that LAMP5-AS1 plays an important role in the self-renewal program and differentiation block in *MLL* leukemia cells [24]. Blockade of self-renewal program and inducing cell death are two major indicators for evaluating the potential applicability of various methods in leukemia treatment [25–27]. However, it remains unknown whether LAMP5-AS1 also regulates apoptosis in *MLL* leukemia cells and whether it could serve as a promising therapeutic target for *MLL* leukemia by affecting multiple carcinogenic pathways. Recent developments suggest that mediating the degradation of MLL or MLL fusion proteins may be a selective strategy in the treatment of *MLL* leukemia [28–30]. We asked if LAMP5-AS1 could couple with MLL fusion proteins and involve in fusion protein degradation pathway and regulates *MLL* leukemia cell survival?

In this regard, we explored lncRNA LAMP5-AS1 functions as a crucially antiapoptotic factor for *MLL* leukemia. Knockdown of LAMP5-AS1 can significantly induce *MLL* leukemia cell apoptosis. LAMP5-AS1 interacts with DOT1L to directly activate the expression of LAMP5, which, in turn, regulates the autophagic degradation of MLL fusion proteins. This novel lncRNA-DOT1L-LAMP5 axis represses the degradation of MLL fusion proteins and sustains the survival of *MLL* leukemia cells. Our findings suggest that targeting LAMP5-AS1 may hold promise as a therapeutic strategy for *MLL* leukemia.

## Methods

### Cell isolation and culture

Bone marrow samples were collected from four *MLL* leukemia patients at the time of their initial diagnosis, with informed consent obtained from patients treated at the First Affiliated Hospital of Sun Yat-sen University. Ethical approval for sample collection was granted by the Hospital’s Protection of Human Subjects Committee, and detailed clinicopathological characteristics of the patients can be found in Additional file 1: Table S1.

Human THP1 and MOLM-13 cells (ATCC, USA) were cultured in RPMI-1640 medium (HyClone, USA), while MV4-11 cells (ATCC, USA) were cultured in IMDM (HyClone, USA). All cell cultures were supplemented with 10% fetal bovine serum (HyClone, USA) and maintained at 37 °C in a 5% CO2 atmosphere. The primary cells cultured in IMDM (HyClone) supplemented with 20% FBS.

### RNA isolation and quantitative real-time PCR (RT-PCR)

Total RNA was extracted from bone marrow and cell samples using an Invitrogen™ TRIzol™ Kit (Thermo Fisher, USA) according to the manufacturer’s instructions and was stored at -80 °C before RT-PCR. RNA was reverse-transcribed into cDNA with the PrimeScript® RT reagent Kit (Takara, DDR047A, Japan). Quantitative RT-PCR for lncRNA and mRNA was performed using the SYBR Premix ExTaq real-time PCR Kit (Takara, Japan) according to the manufacturer’s instructions. All of the data were normalized to GAPDH expression as a control. The expression level for each lncRNA and mRNA was determined using the 2^−△△Ct^ method. All primers were confirmed by sequencing the PCR product fragments, as shown in Additional file 1: Table S2.

### RNA interference and lentivector expression systems

RNA interference using siRNA (Ribobio, China) was performed to knockdown LAMP5-AS1 and LAMP5. Transient transfections of siRNAs were performed using the Neon Transfection System (Invitrogen, USA) with 10 µl reactions according to the manufacturer’s guidelines. Lentiviral shRNA templates were cloned into the pGreenPuro™ shRNA Cloning and Expression Vector (System Biosciences, Germany), and pSIH1-H1-siLuc-copGFP was used as negative control. Tandem mRFP-GFP-LC3 plasmid for autophagy flux was constructed as previously described [31–33]. For stable expression assays, lentiviral pGreenPuro™ shRNA vectors and pCDH-CMV-MCS-EF1-Puro eukaryotic expression vector were packaged into lentiviruses using Lentivector Expression Systems (System Biosciences, Germany) consisting of pPACKH1-GAG, pPACKH1-REV, and pVSV-G. Finally, the lentiviruses were transformed into THP1, MV4-11 and MOLM-13 cells, and the transformed cells were then selected with puromycin. The primers for siRNAs and shRNAs were described in shown in Additional file 1: Table S2.

### Protein extraction and immunoblotting

Total protein was extracted from bone marrow samples and cells using RIPA lysis buffer (Beyotime Biotechnology, China) with 1× Thermo Scientific™ Halt™ Protease Inhibitor Cocktail (Thermo Fisher, USA) according to the manufacturer’s instructions. For tumor samples, the tumor cells were first homogenized from the tumor tissues by a freezing grinder (LUKYM-II, China), and then, the total protein was extracted from the tumor cells.

For Immunoblotting, all protein samples were suspended in 5x loading buffer and then denatured for 5 min at 100 °C, separated via SDS-PAGE, transferred to PVDF membranes, and blotted. The antibodies are as fellow: MLL1 (BETHYL, A300-086 A-3); LC3B (Sigma, L7543); LAMP5(Sigma, A78501); β-tubulin (Invitrogen, 32-2600); β-actin (Sigma, A2228); GAPDH (Proteintech, 10494-1-AP); DOT1L (CST, 90,878 S); H3(Abcam, Ab1791); H3K79me2(Abcam, Ab3594); H3K79me3(Abcam, Ab2621); IgG (CST, 3900); Goat Anti-Mouse IgG HRP (Thermo Fisher, H10007); Goat Anti-Rabbit IgG HRP (Thermo Fisher, A18903); Goat Anti-Rabbit IgG H&L DyLight® 594(Abcam, ab96885);Goat Anti-Rabbit IgG H&L DyLight® 647 (Abcam, ab150115).

### RNA pull-down

We performed pull-down assays as previously described [24, 34]. Pierce™ Magnetic RNA-Protein Pull-Down Kit (Thermo Fisher, USA) was used to perform the pull-down assays with the biotinylated LAMP5-AS1 and LAMP5-AS1 antisense. Finally, the proteins enriched by the biotinylated LAMP5-AS1 and LAMP5-AS1 antisense were resolved via SDS-PAGE and western blotting.

### Chromatin immunoprecipitation

ChIP analysis is performed on chromatin extracts from THP1 and MOM-13 cells using a Magna ChIP™ G-Chromatin Immunoprecipitation Kit (17–611) (Merck Millipore, Germany) with dimethyl- and trimethyl-Histone H3 (Lys79) according to the manufacturer’s standard protocol. Rabbit IgG served as the negative control. The fold-enrichment of H3K79me2/3 was quantified by quantitative RT-PCR and calculated relative to the input chromatin. The primers used for ChIP-qPCR analysis were listed in Additional file 1: Table S2.

### RNA and DNA fluorescence in situ hybridization (FISH) and DOT1L immunofluorescence

To detect the subcellular location of LAMP5-AS1 RNA on *LAMP5* locus, we carried out FISH in THP1 cells using the RiboTM Fluorescent In Situ Hybridization Kit (RiboBio, China). Cells were washed briefly with PBS and then fixed in 4% formaldehyde for 15 min at room temperature. Cells were permeabilized in PBS containing 0.5% Triton X-100 on ice for 5 min and then blocked in the preliminary hybridization solution for 30 min at room temperature after three washes with PBS for 10 min each. Hybridization was carried out using the Cy3-labeling LAMP5-AS1 FISH Probe Mix (RiboBio, China) and Digoxigenin (DIG) -labeling *LAMP5* DNA primers (the DNA primers were labeled DIG using the DIG High Prime DNA Labeling and Detection Starter Kit II, Roche, Switzerland) in a humidified chamber at 37 °C for 12–16 h. Cells were rinsed with SSC buffer in accordance with the order 4×, 2×, and 1×. For co-localization studies, after RNA and DNA FISH, cells were fixed again for 5 min in 2% formaldehyde and subjected to immunofluorescence with DOT1L primary antibody (CST, 90,878 S) and followed by DIG-FITC (Abcam, ab119349) and Goat Anti-Rabbit IgG H&L DyLight® 647 (Abcam, ab150115). Finally, cells were observed on a Zeiss7 DUO NLO confocal laser microscope (Carl Zeiss, Germany).

### Transmission electron microscopy

TEM images of autophagosomes were obtained from thin sections using a JEM1400 electron microscope (JEOL, Japan). The autophagy fluorescence signals were obtained using an anti-LC3B antibody (Novus Biologicals, Taiwan of China) analyzed by Zeiss7 DUO NLO confocal laser microscope (Carl Zeiss, Germany).

### Chromatin isolation by RNA purification (ChIRP) assay

ChIRP assay was performed as previously described [35, 36]. First, a pool of 13 probes would cover the whole LAMP5-AS1 for achieving the maximal capture ratio. All of the single stranded LAMP5-AS1 DNA probes with 3’-Biotin TEG modification. Random DNA probes with 3’-Biotin as the negative control. A total of 200 million cells were harvested and cross-linked by 3% formaldehyde. After wash by PBS, the cells were lysed by the lysis buffer (with fresh Protease Inhibitor, PMSF, and RNasin) to each tube and resuspend the pellet (avoid clumps), proceed immediately to sonication. The samples were added the hybridization buffer and followed by ChIRP procedure. Finally, the RNA, DNA and protein are eluted and detected.

### Flow cytometric analysis

For cell apoptosis assay, cells were stained with Annexin V/FITC and propidium iodide (PI) (Lianke, China) and then analyzed by flow cytometry (BD, USA). For GFP positive cells in mice samples, cells were first separated from mice organs such as blood, bone marrow, spleen and liver. These mice-derived cells were washed with cooling PBS and then were analyzed on a BD FACSCelesta analyzer (BD Biosciences, USA).

### Animal model

The xenotransplantation experiments were performed as previously described [24, 37, 38]. Five-week-old male NOD/SCID mice were maintained under specific pathogen-free conditions in the Laboratory Animal Center of Sun Yat-sen University. All experimental procedures were performed according to the institutional ethical guidelines for animal experiments. Mice were randomly assigned to three groups of ten mice each. In each group, lentiviral stably transduced sh-NC, sh-LAMP5-AS1 (2 × 10^6^ MOLM-13) were subcutaneously injected into the mice; while 2 × 10^6^ MV4-11 cells transduced sh-NC, sh-LAMP5-AS1 were intravenously (tail vein) implanted mice. For subcutaneously implanted mice, tumor growth was monitored for 2 days each. Three weeks after inoculation, the intravenously implanted mice were sacrificed for analysis. Human cell engraftment (GFP + cell populations) in the bone marrow, peripheral blood, spleen, and liver was evaluated by flow cytometry. The remaining mice were performed the survival assay.

### Statistical analysis

Spearmen and Pearson’s correlation coefficient was used to determine the correlation between LAMP5-AS1 and LAMP5 expression. Fisher’s exact test was used to determine the significance of two groups. Mean ± SEM was used to analyzed the three independent experiments. Two-tailed tests were used for univariate comparisons. Kaplan-Meier method with a log-rank test is used to analyze the mice survival. *p* < 0.05 was considered statistically significant.

## Results

### Targeting LAMP5-AS1 decreases MLL fusion protein and induces *MLL* leukemia cell apoptosis

**Fig. 1:**
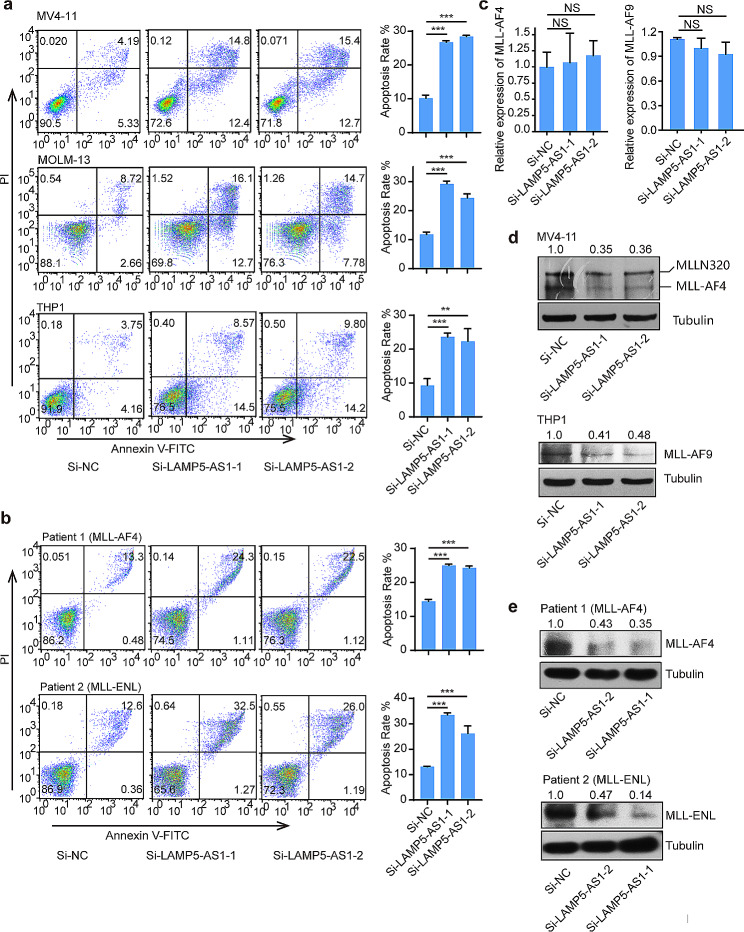
Targeting LAMP5-AS1 induces *MLL* leukemia cell apoptosis and decreases MLL fusion protein. (**a**, **b**) Cell apoptosis was measured by flow cytometry in both *MLL* leukemia cell lines (MV4-11, MOLM-13 and THP1) (**a**) and two primary cells from patients with *MLL* leukemia (**b**) transfected with LAMP5-AS1 siRNA and the control. Error bars reflect ± SEM (**, *p* < 0.01, ***, *p* < 0.001, right) in three independent experiments. (**c**) Quantitative RT-PCR for MLL-AF4 (MV4-11) and MLL-AF9 (THP1) under knockdown of LAMP5-AS1. NS: not significant. (**d, e**) Western blotting was performed to detect MLL fusion protein levels in cell lines (**d**) and primary cells (**e**) when LAMP5-AS1 was knocked down. The MLL-AF9, -AF4 or -ENL/ Tubulin densitometric ratio was recorded by ImageJ

Previous study has shown that lncRNA LAMP5-AS1 is the most highly differentially expressed lncRNA in *MLL* leukemia patients and play a role in the self-renewal program [24]. To ascertain the potential of LAMP5-AS1 as a therapeutic target for *MLL* leukemia, we explored the function of LAMP5-AS1 on *MLL* leukemia cell survival. The silencing of LAMP5-AS1 resulted in a marked increase in apoptosis, indicating its oncogenic function in *MLL* leukemia (Fig. 1a and Supplementary Fig. 1a). We further investigated the anti-apoptosis role of LAMP5-AS1 in primary *MLL* leukemia cells that obtained from two different patients. The results, as depicted in Fig. 1b and Supplementary Fig. 1b, demonstrated a significant elevation in apoptosis rates following the knockdown of LAMP5-AS1. This finding highlights the role of LAMP5-AS1 as a critical antiapoptotic factor and underscores its importance as a therapeutic target in *MLL* leukemia. Previous research also established a strong positive correlation between the expression of LAMP5-AS1 and MLL fusion protein levels, suggesting a regulatory role in cell survival via the maintenance of MLL fusion proteins [24]. In line with this hypothesis, our study reveals that altering LAMP5-AS1 expression levels minimally affects the mRNA levels of MLL fusion proteins but significantly reduces their protein levels (Fig. 1c-e). These results suggest that LAMP5-AS1 influences *MLL* leukemia cell survival primarily through the modulation of MLL fusion protein levels. Collectively, our findings reinforce the concept that LAMP5-AS1 modulates MLL fusion protein levels, thus promoting leukemia cell survival, and present a compelling case for its role as a potential therapeutic target in *MLL* leukemia.

We further performed in vivo experiment in a NOD/SCID xenograft mouse model. We transfected MV4-11 cells with LAMP5-AS1 short hairpin RNA (shRNA) and a control shRNA (referred to as sh-NC) (Supplementary Fig. 1c).

**Fig. 2:**
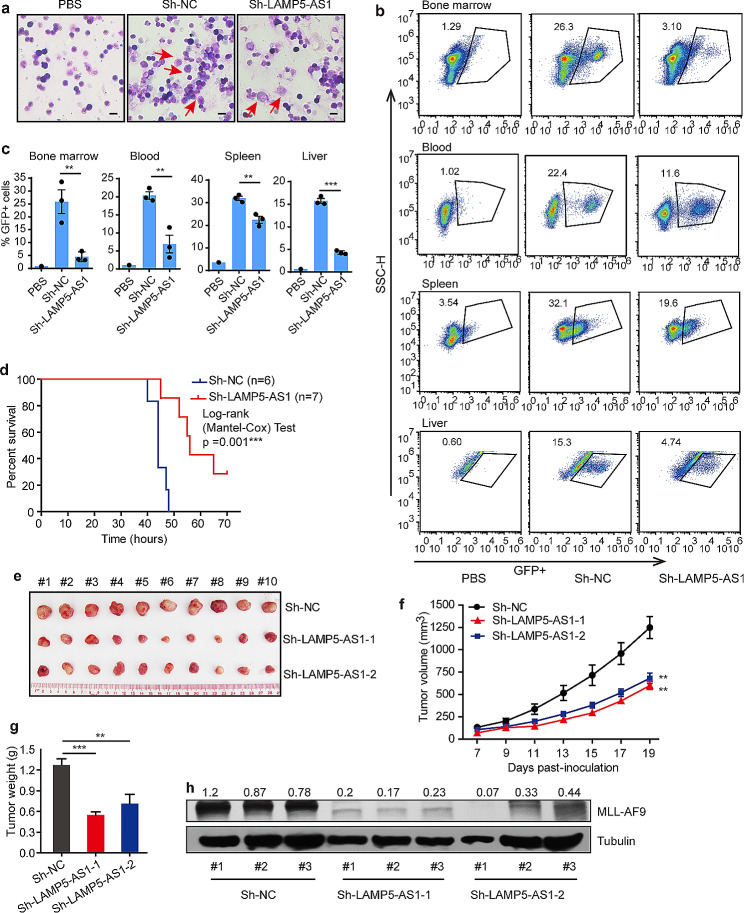
Knocking down LAMP5-AS1 suppress MLL fusion protein expression and infiltration of *MLL* leukemia in vivo. (**a**) Wright-Giemsa staining of bone marrow of mouse recipients of PBS, sh-NC cells or sh-LAMP5-AS1 MV4-11 cells. MV4-11 cells were indicated by the red arrows. Scale bars, 50 μm. (**b**) Representative flow cytometry graphs showing the blasts in organ samples from mice treated with sh-NC or sh-LAMP5-AS1 knockdown MV4-11 cells. Mice treated with PBS were used as the negative controls. (**c**) Histogram plots show the statistical values for (**b**) Error bars reflect ± SEM (**, *p* < 0.01, ***, *p* < 0.001). **d** Kaplan–Meier curves show the survival of mice intravenously injected with sh-NC and sh-LAMP5-AS1 MV4-11 cells (***, *p* < 0.001). Following subcutaneous inoculation of shRNA-transformed MOLM-13 cells into the flanks of NOD-SCID mice, LAMP5-AS1 knockdown inhibited the malignant proliferation of *MLL* leukemia cells and subsequent tumor size (**e**), growth (**f**), and weight (**g**) in vivo. Error bars reflect ± SEM (ten mice per group, **, *p* < 0.01; ***, *p* < 0.001). (**h**) MLL-AF9 protein levels dramatically decreased in LAMP5-AS1-knockdown MOM-13 cell-transfected NOD/SCID mice. The MLL-AF9/ Tubulin densitometric ratio was recorded by ImageJ

These cells were subsequently intravenously implanted into NOD/SCID mice. Three mice of each group were randomly culled after 3 weeks and the infiltration of cancer cells were assessed. The success of mouse model establishment was confirmed by appearance of MV4-11 cells in bone marrow (Fig. 2a). We found that the percentages of GFP + cells were decreased in the bone marrow, peripheral blood, spleen, and liver, of the mice treated with the sh-LAMP5-AS1 MV4-11 cells compared with those in the animals treated with sh-NC-transfected cells (Fig. 2b and c). Specifically, the sh-LAMP5-AS1 groups survived longer than the control groups (Fig. 2d), suggesting that the knockdown of LAMP5-AS1 could effectively inhibit the malignant progression of *MLL* leukemia. Additionally, we developed a NOD/SCID xenograft mouse model with subcutaneously transfected MOLM-13 cells, also targeting LAMP5-AS1. As depicted in Fig. 2e, the shRNA-mediated knockdown of LAMP5-AS1 markedly inhibited the malignant proliferation of *MLL* leukemia-positive tumors. Furthermore, we observed a significant decrease in both tumor growth and weight in the treated group (Fig. 2f-g). A concurrent reduction in MLL-AF9 protein levels was also noted in the sh-LAMP5-AS1 transfected NOD/SCID mice (Fig. 2h). LAMP5-AS1-mediated pathogenesis suggested that targeting LAMP5-AS1 may be a selective strategy for *MLL* leukemia treatment.

### LAMP5-AS1 actives LAMP5 expression

**Fig. 3:**
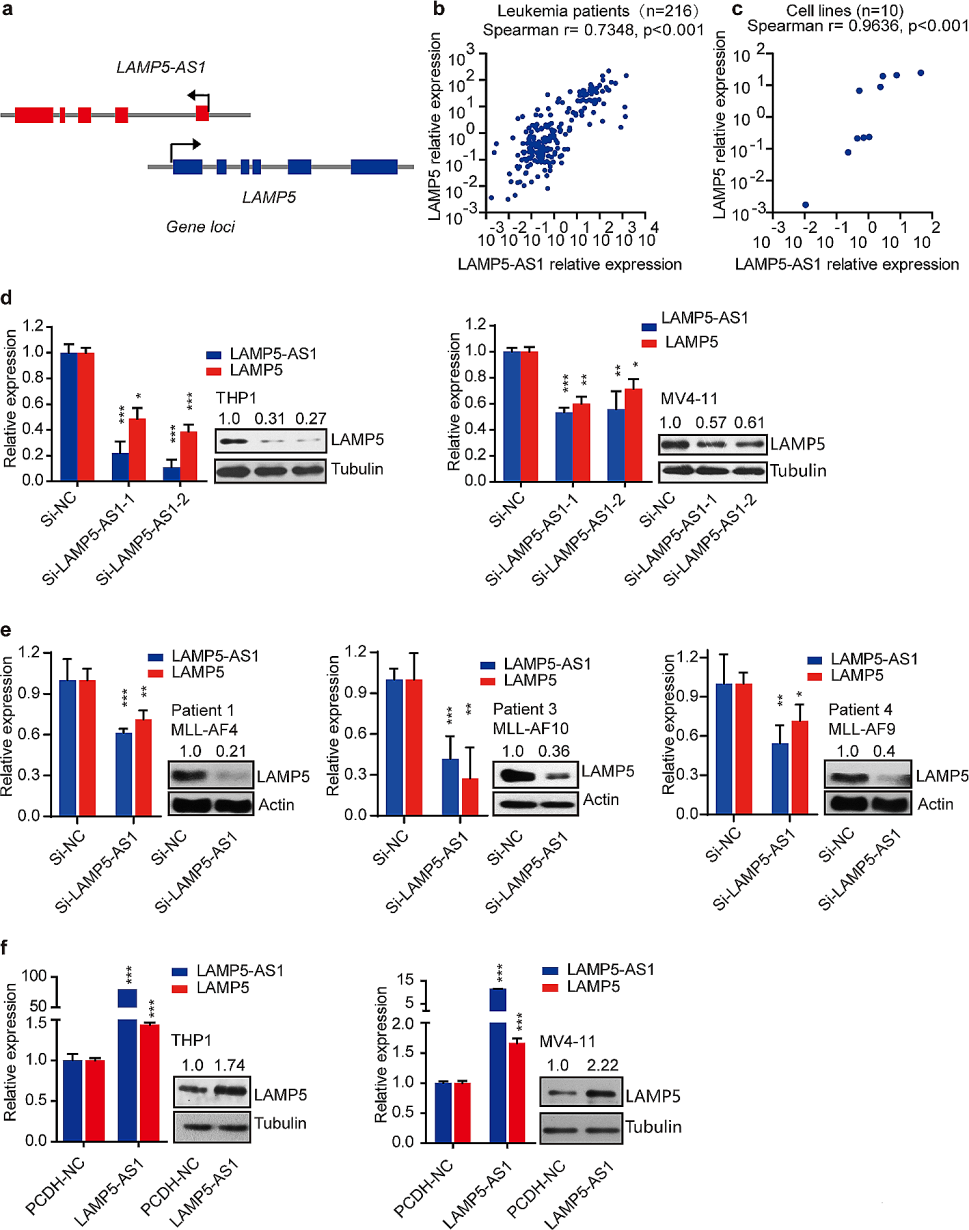
LAMP5 expression level is regulated by LAMP5-AS1. (**a**) Schematic depiction of *LAMP5-AS1* and *LAMP5* gene loci. (**b, c**) The co-expression of LAMP5-AS1 and LAMP5 was validated in leukemia patient samples (**b**) and leukemia cells(**c**). Spearman method was used to analyze the correlation coefficient. (**d, e**) Quantitative RT-PCR and western blotting show that Knocking down LAMP5-AS1 by siRNAs reduce LAMP5 expression in *MLL* leukemia cell lines (**d**) and primary cells (**e**). Error bars reflect ± SEM (*, *p* < 0.05; **, *p* < 0.01; ***, *p* < 0.001) from three independent experiments. (**f**) Lentiviral overexpression of LAMP5-AS1 increased LAMP5 expression in *MLL* leukemia cells. Error bars reflect ± SEM (*, *p* < 0.05; **, *p* < 0.01; ***, *p* < 0.001) from three independent experiments. The LAMP5/ Tubulin densitometric ratio was recorded by ImageJ

We next sought to determine how the lncRNA LAMP5-AS1 regulates *MLL* leukemia cell survival via maintaining MLL fusion protein levels. Upon reanalyzing the co-expression pattern of differentially expressed lncRNAs and mRNAs in our previous RNA-seq data [24, 39], we identified a striking correlation between LAMP5-AS1 and a lysosome-associated membrane protein, LAMP5, which is the antisense counterpart of LAMP5-AS1 (Fig. 3a and Supplementary Fig. 2a). We found that the protein level of LAMP5 is also high in *MLL* leukemia patients and further verified the positive correlation of LAMP5 and LAMP5-AS1 expression in various datasets, including *MLL* leukemia patient samples and cell lines, confirming their association (Fig. 3b, c and Supplementary Fig. 2b-d). Interestingly, combination of LAMP5-AS1 and LAMP5 expression can significantly discriminate the *MLL*-rearranged (*MLL*-r) from *MLL*-wt leukemia sets (Supplementary Fig. 2e). Previous study has revealed that LAMP5 can prevent MLL fusion proteins from undergoing autophagic degradation, suggesting its functional relevance in *MLL* leukemia progression or maintenance. Thus, we hypothesized that LAMP5-AS1 maintains MLL fusion proteins by targeting LAMP5.

To test this hypothesis and determine whether LAMP5 is indeed a downstream effector of LAMP5-AS1 in regulating MLL fusion proteins, we examined the effect of LAMP5-AS1 on LAMP5 expression by modulating the expression of LAM5-AS1. As shown in Fig. 3d and Supplementary Fig. 3a, knocking down LAMP5-AS1 significantly reduce LAMP5 expression both in mRNA and protein levels. This effect was consistently observed in patient primary cells (Fig. 3e). In addition, overexpressing LAMP5-AS1 can considerably enhance the expression of LAMP5 both in mRNA and protein levels (Fig. 3f and Supplementary Fig. 3b). However, LAMP5-AS1 expression levels did not significantly change when LAMP5 was knocked down (Supplementary Fig. 3c-e). These observed above suggests that LAMP5 functions as the downstream effector of LAMP5-AS1.

### LAMP5-AS1 interacts with DOT1L to enhance H3K79 methylation at the *LAMP5* locus

**Fig. 4:**
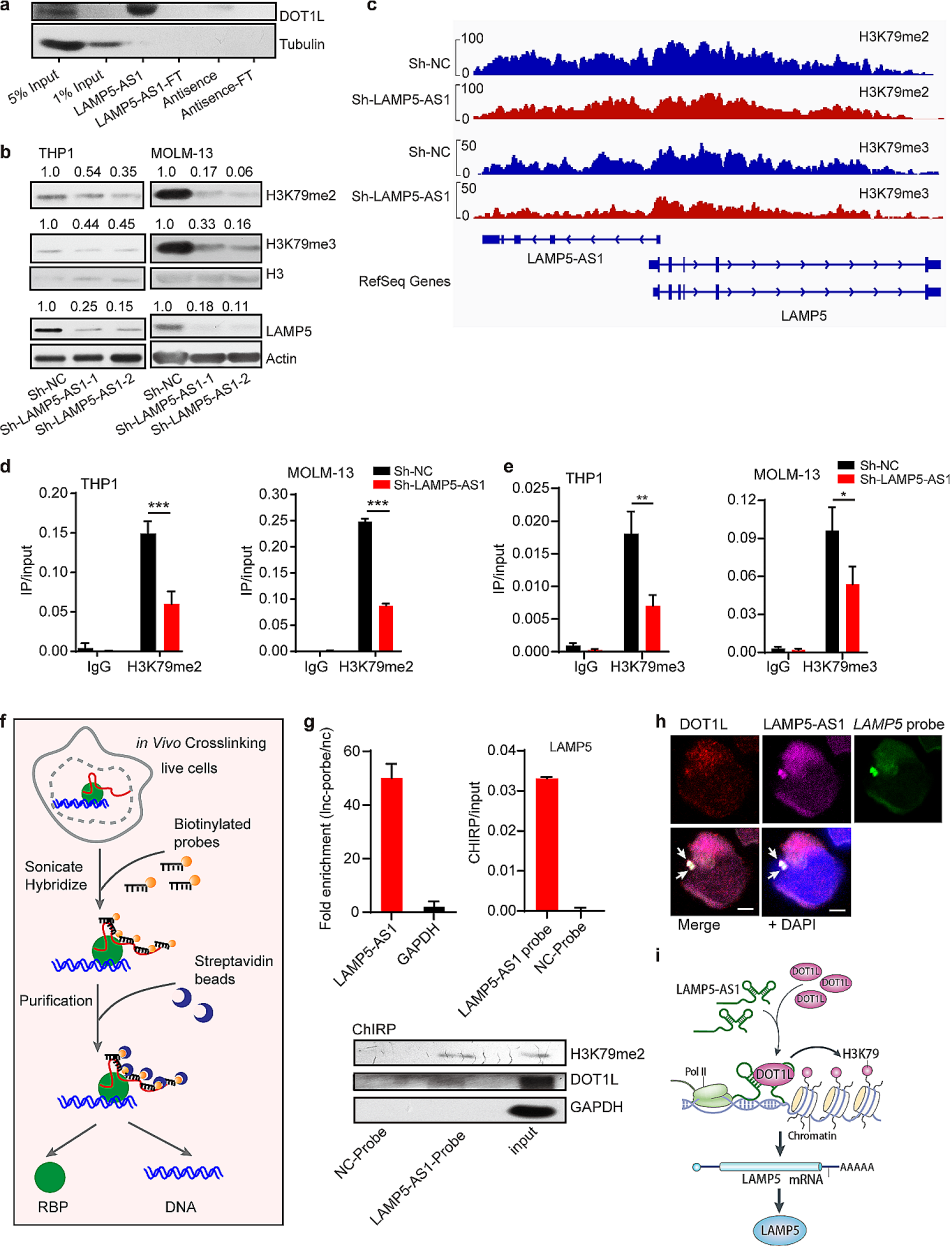
LAMP5-AS1 regulates LAMP5 expression through the binding of DOT1L to the *LAMP5* locus. (**a**) Western blotting show that DOT1L was enriched by LAMP5-AS1 pull-down assays. Antisense of LAMP5-AS1 sequence indicates the negative control. FT: flow through. (**b**) Western blotting for the protein levels of H3K79me2, H3K79me3, and LAMP5 in *MLL* leukemia cells transduced by LAMP5-AS1 siRNA and control. H3 and beta-actin as the internal control. The H3K79me2 or H3K79me3/ H3, LAMP5/Actin densitometric ratio were recorded by ImageJ, respectively. (**c**) ChIP-seq profiles (shown in GSE150483) of H3K79me2 and H3K79me3 at the *LAMP5* genomic loci in LAMP5-AS1 knockdown (red) compared with control (blue) MOLM-13 cells. The y-axis scales represent read density per million sequenced reads. (**d, e**) ChIP Quantitative RT-PCR assays show H3K79me2 (**d**) and H3K79me3 (**e**) on the *LAMP5* gene locus clearly declined upon LAMP5-AS1 knockdown in *MLL* leukemia cells. Error bars reflect ± SEM (**, *p* < 0.01; ***, *p* < 0.001) from three independent experiments. (**f**) Diagram depicts the procedure of ChIRP. (**g**) Quantitative RT-PCR for the LAMP5-AS1(upper, left) and *LAMP5* locus (upper, right) enrichment in ChIRP experiment captured by LAMP5-AS1 probes. GAPDH and NC-probe were used to be the negative control. Western blotting shows the DOT1L and H3K79me2 protein levels that captured by LAMP5-AS1 probes in ChIRP experiment. GAPDH protein as the negative control. (**h**) RNA and DNA FISH and IF experiments showed that LAMP5-AS1 and DOT1L co-localized at LAMP5 locus in the cell nucleus. (**i**) Proposed model for LAMP5-AS1 regulate LAMP5 in *MLL* leukemia. LAMP5-AS1 regulates LAMP5 by directly interacting with DOT1L

We next explore the underlying mechanism by which LAMP-AS1 regulates LAMP5. Previous study has demonstrated that LAMP5-AS1 modulates the methyltransferase activity of DOT1L, leading to the enhancement of global patterns of H3K79 methylation at gene loci [24]. Importantly, *LAMP5* was regulated by DOT1L, with an enrichment of H3K79 located at its genetic locus [29]. Therefore, we hypothesized that LAMP5-AS1 might directly interact with DOT1L to regulate LAMP5 expression. The result has shown that LAMP5-AS1 directly interacts with DOT1L, and knocking down LAMP5-AS1 led to a significant decrease in both H3K79 di-/tri-methylation and LAMP5 expression levels (Fig. 4a and b), suggesting that LAMP5-AS1 could regulate the LAMP5 expression by affect H3K79-methylated state in its gene locus. As expected, when analyzing the locus of *LAMP5* in the GSE150483 dataset, we observed a substantial reduction in H3K79 di-/tri-methylation in sh-LAMP5-AS1 cells compared to control cells (Fig. 4c). The ChIP assays further confirmed the downregulation of H3K79 di-/tri-methylation at the LAMP5 gene body upon LAMP5-AS1 knockdown (Fig. 4d and e).

To gain a comprehensive understanding of the interactions among LAMP5-AS1, DOT1L, and the LAMP5 locus, we conducted a ChIRP assay (Fig. 4f). As a result, LAMP5-AS1 was significantly enriched by the label probes (Fig. 4g). Moreover, both typical DNA sequence for *LAMP5* and DOT1L/ H3K79me2 were captured by LAMP5-AS1 (Fig. 4g). Specifically, FISH and immunofluorescence experiments also revealed that LAMP5-AS1 co-localized with both DOT1L protein and the LAMP5 locus within the cell nucleus (Fig. 4h), illustrating that LAMP5-AS1 can feasibly interact with DOT1L in the *LAMP5* locus. Moreover, we found that knocking down DOT1L can also reduce LAMP5-AS1 expression, implying that DOT1L may regulate LAMP5-AS1 expression and then affect the recruitment of LAMP5-AS1/DOT1L complex to the *LAMP5* gene loci (Supplementary Fig. 3f). These results together demonstrated that LAMP5-AS1 regulates its downstream effector LAMP5 by directly interacting with DOT1L to deposit activating H3K79 markers (Fig. 4i).

### LAMP5-AS1-LAMP5 axis regulates autophagy and autophagic degradation of MLL fusion proteins

**Fig. 5:**
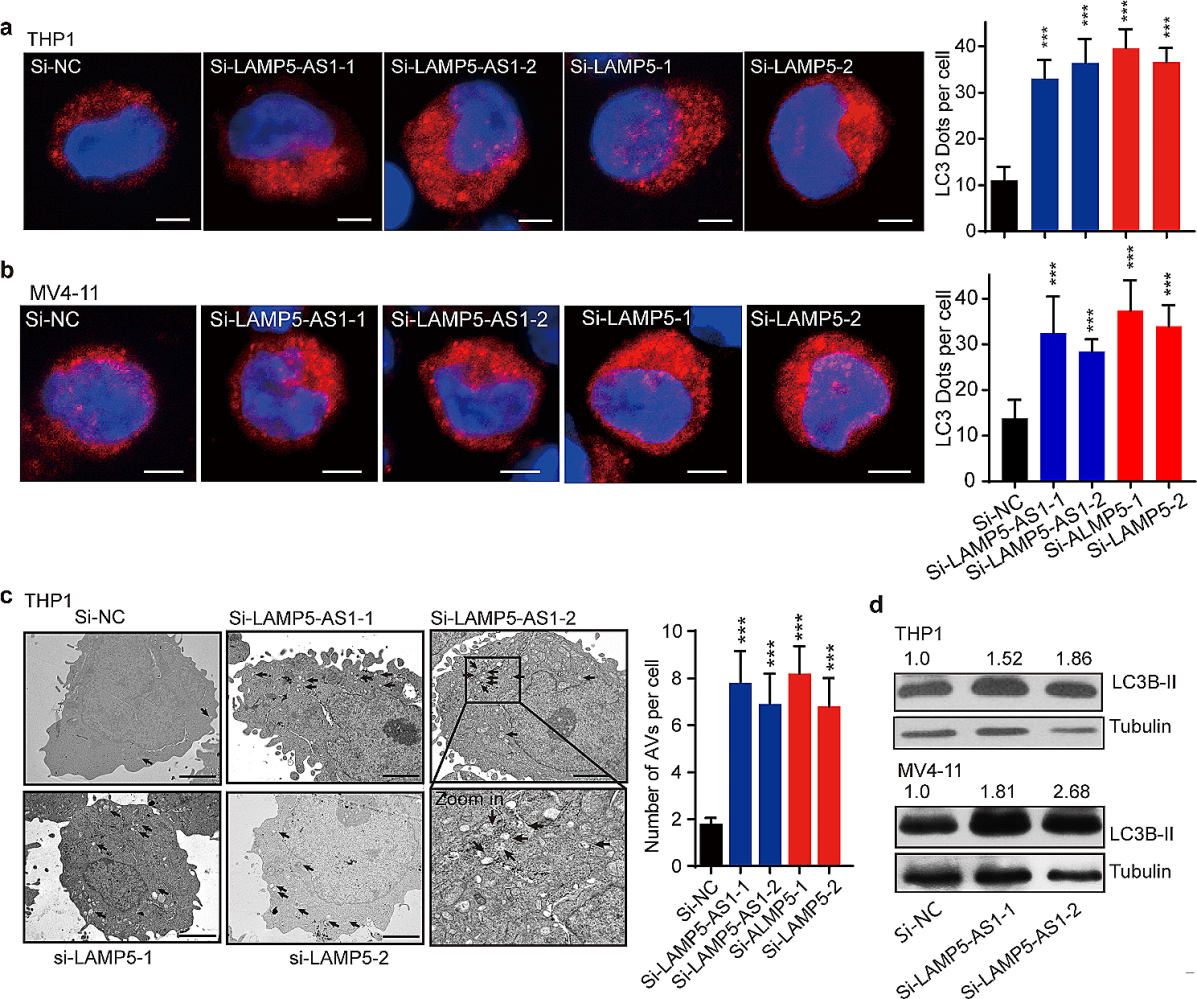
LAMP5-AS1- LAMP5 axis regulate cell autophagy. (**a, b**) Representative IF graphs (left) showing LC3B puncta accumulation in THP1(**a**) and MV4-11(**b**) cells. Histogram plots show the statistical values for the number of LC3B puncta per cell calculated by Image-Pro Plus (right, *n* = 20 cells; Error bars reflect ± SEM, ***, *P* < 0.001). Scale bar, 10 mm. (**c**) Representative TEM graphs (left) showing autolysosomes (indicated by arrows) in THP1 cells. Autolysosomes accumulated in the LAMP5-AS1- or LAMP5-downregulated cells than those in si-NC samples. Error bars reflect ± SEM (ten cells per group, ***, *p* < 0.001). (**d**) Western blotting showing LC3B-II enrichment after LAMP5-AS1 or LAMP5 knockdown in THP1 and in MV4-11 cells. beta-tubulin as the internal control. The LC3B-II /Tubulin densitometric ratio were recorded by ImageJ

Based on the previous observations confirming LAMP5 as the direct downstream effector of LAMP5-AS1, we explored whether reducing LAMP5-AS1 levels could decrease MLL fusion proteins by modulating LAMP5 expression within the autophagy pathway. To investigate the physiological relevance of LAMP5 induction by LAMP5-AS1 in autophagy, we employed three canonical assays [31]. Firstly, the autophagy-related marker LC3B was evaluated. The results showed significant LC3B puncta accumulation in both LAMP5-AS1 and LAMP5-downregulated *MLL* leukemia cells than those in negative controls (Fig. 5a and b and Supplementary Fig. S4a). Next, we conducted ultrastructural analysis of autolysosome formation using transmission electron microscopy (TEM). As indicated by arrows in Fig. 5c, autolysosome formation was notably increased in cells with downregulated LAMP5-AS1 or LAMP5 (Fig. 5c). Thirdly, we assessed LC3B cleavage and turnover (LC3B-II) protein levels, which also showed that knocking down LAMP5-AS1 enhanced LC3B-II expression in *MLL* leukemia cell lines (Fig. 5d and Supplementary Fig. S4b). The results clearly showed that autophagy was enhanced when either LAMP5-AS1 or LAMP5 was knocked down.

**Fig. 6:**
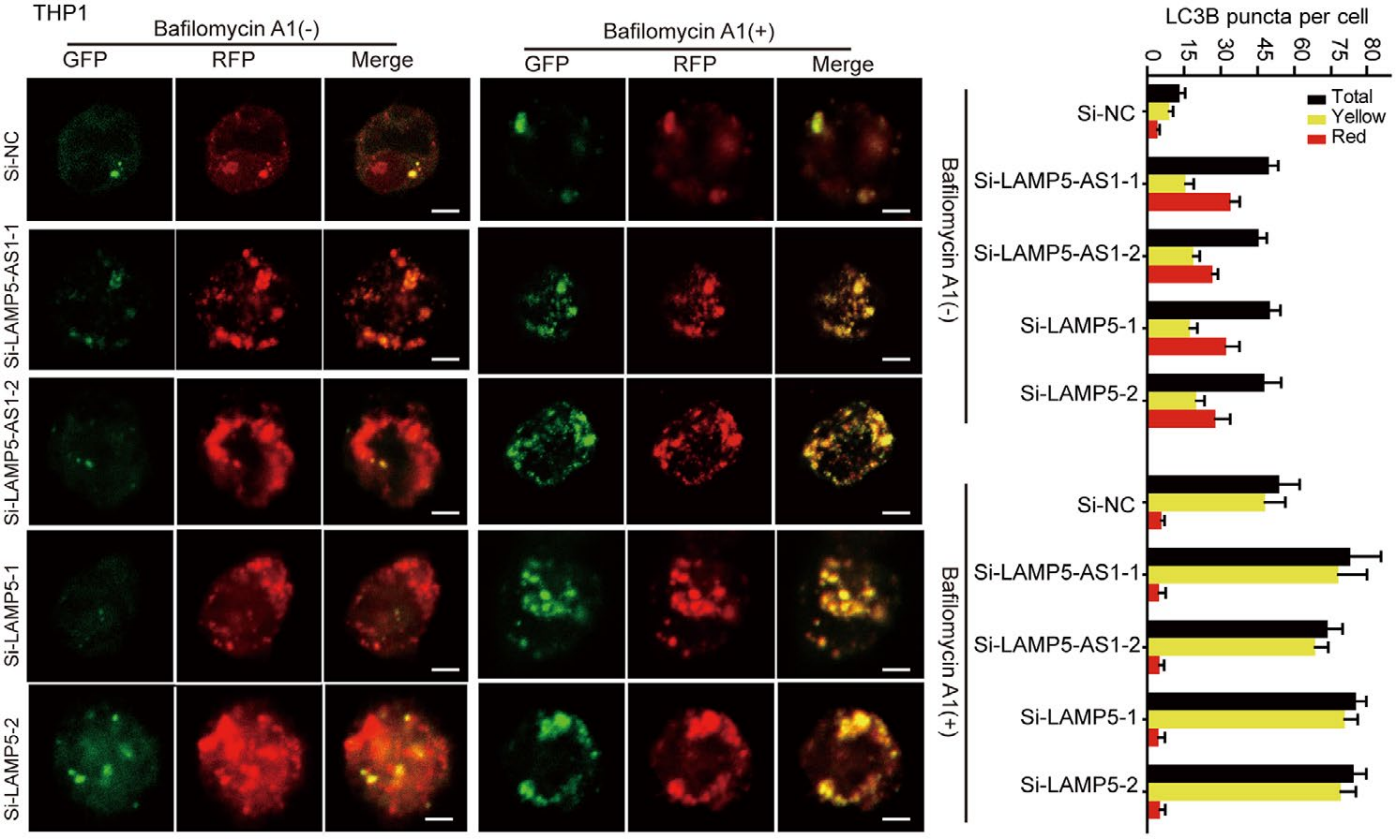
LAMP5-AS1-LAMP5 axis regulates autophagic flux. siRNAs targeting LAMP5-AS1 and LAMP5 were transient transfected into stable mRFP-GFP-LC3 THP1 cells which were treated with or without bafilomycin A1 (25 nM, 12 h). Scale bar, 10 μm. The numbers of yellow LC3 puncta and red LC3 puncta per cell in each condition were quantified using Image-Pro Plus. Total LC3 puncta represent the number of yellow LC3 puncta and red LC3 puncta. More than 20 cells were counted in each condition (Mean ± SEM)

**Fig. 7:**
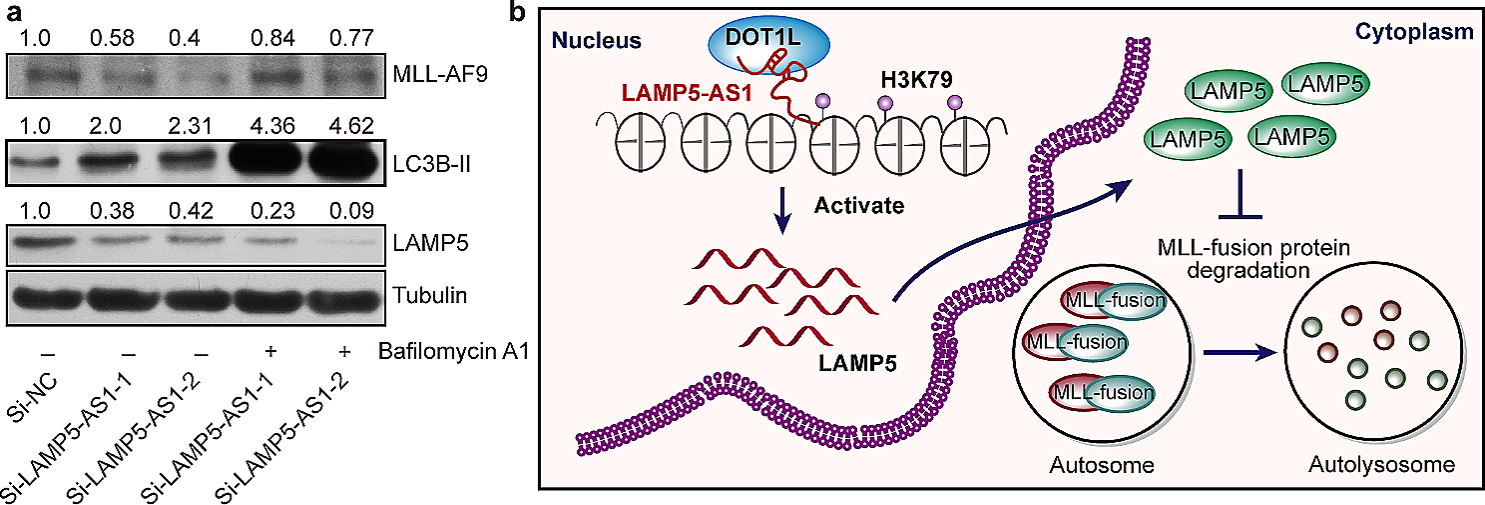
The LAMP5-AS1-DOT1L-LAMP5 axis suppresses autophagy and maintains MLL fusion protein. (**a**) The resulting decline in MLL-AF9 levels by knocking down either LAMP5-AS1 was reversed upon treatment with the autophagy inhibitor bafilomycin A1 (25 nM, 12 h). MLL-AF9, LC3B-II, and LAMP5 were assayed by western blotting, and beta-tubulin as a loading control. The MLL-AF9, LC3B-II, or LAMP5 /Tubulin densitometric ratio were recorded by ImageJ. (**b**) Proposed model for the LAMP5-AS1-DOT1L-LAMP5 axis regulates autophagy and maintains MLL fusion protein

We subsequently performed fluorescence analysis of tandem mRFP-GFP-LC3 [31, 33], in which exogenous LC3B is expressed using a lentivirus system, to validate whether both LAMP5-AS1 and LAMP5 regulate autophagic flux in *MLL* leukemia cells. The results clearly showed that the accumulation of mRFP-LC3B puncta and yellow puncta (caused by the increased pH by Bafilomycin A1 in autolysosomes [31]) per cell significantly increased in cells transfected with siRNAs against LAMP5-AS1 or LAMP5 compared with the negative control cells (Fig. 6 and Supplementary Fig. 5). Taken together, these data suggest that LAMP5-AS1 suppresses autophagy in *MLL* leukemia cells. To further demonstrate that the effect of LAMP5-AS1-mediated LAMP5 on the degradation of MLL fusion protein occurs via autophagy, we treated the cells with autophagy inhibitor, bafilomycin A1 [31]. We observed that knocking down LAMP5-AS1 significantly downregulated MLL-AF9 protein levels, and this decrease was reversed upon treatment with the autophagy inhibitor bafilomycin A1 (Fig. 7a).

In summary, we characterized the lncRNA LAMP5-AS1 associated with *MLL* leukemia cell survival. Importantly, we have elucidated the molecular mechanisms by which LAMP5-AS1 regulates the degradation of MLL fusion proteins. By interacting with DOT1L, LAMP5-AS1 regulates LAMP5, a newly identified critical autophagy-suppressing protein. Thus, we propose a novel lncRNA-DOT1L-LAMP5 axis for the autophagic degradation of MLL fusion proteins as shown in Fig. 7b.

## Discussion

Understanding the regulation and degradation of oncogenic fusion proteins could revolutionize targeted therapy in cancers with unfavorable outcomes [28–30, 40, 41]. It has been reported that chromosomal rearrangements that involve transcriptional regulators occur in 38% of hematological malignancies and in 44% of solid tumors that have abnormal karyotypes [1, 2]. The degradation of fusion oncoproteins via proteolysis-targeting-chimeras (PROTACs) method is useful for lab studies, but this method is very difficult to be druggable in cancer treatment [30, 42–44]. Thus, these types of fusion proteins are generally deemed undruggable [1, 45–47], and the therapeutic options remain largely under developed. Although lncRNAs have recently been demonstrated as key regulators in cancer progression through the regulation of protein expression [14–16, 48, 49], whether lncRNAs are associated with fusion proteins involving transcription factors is not well declared. We previous identified a lncRNA LAMP5-AS1, which shows a specifically higher expression in *MLL* leukemia patients than those without rearrangement and is associated with self-renewal program [24]. In this study, our findings reveal that LAMP5-AS1 plays a pivotal role in the autophagy pathway and can regulate the autophagic degradation of MLL fusion proteins. As MLL fusion proteins have been reported to resist proteasomal degradation due to the lack of a specific binding domain [28, 50], the findings in this study offer an alternative approach for intervention for this type of oncoprotein, which is associated with unfavorable outcomes. Targeting the components of the degradation pathway may be able to efficiently eliminate the fusion protein [51–53]. Moreover, our results shed light on alternative means by which to disturb fusion gene products involving transcriptional regulators.

Several lncRNAs with unique expression patterns have been reported to play essential roles in various cellular and molecular mechanisms driving carcinogenesis [11, 13, 34, 49]. For example, HOTAIR is frequently overexpressed in breast cancer and has been shown to promote cell growth and migration by modulating multiple signaling pathways, including FGFR and Wnt/catenin pathways; while can also facilitate the invasiveness and metastasis of breast cancer by stimulating gene expression via the targeting of PRC2 and reducing Akt/JNK signaling pathway [18, 19]. Knocking down HOTAIR could offer a selective strategy for breast cancer treatment via repress a variety of carcinogenic pathways simultaneously. We previous study have identified a special upregulation lncRNA LAMP5-AS1 in *MLL* leukemia [24]. LAMP5-AS1 was found to regulate the self-renewal program and differentiation block by enhancing the methyltransferase activity of DOT1L in *MLL* leukemia [24]. In the current study, we provide the first evidence of LAMP5-AS1’s role in *MLL* leukemia cell survival. Knocking down LAMP5-AS1 can induce *MLL* leukemia cell apoptosis via activating an autophagy pathway, which solidly supports LAMP5-AS1 as a therapeutic target for *MLL* leukemia. LAMP5-AS1 inhibition could be a promising strategy for *MLL* leukemia treatment via inducing cell differentiation and autophagic apoptosis simultaneously. Searching for and targeting the pivotal lncRNAs, such as HOTAIR, ANRIAL, MALAT1, and LAMP5-AS1, which are involved in various carcinogenesis, may bring new hope for treatment-refractory patients.

In the current study, we first link a lncRNA to H3K79 methylation for regulating the selective degradation of MLL fusion proteins. We showed that LAMP5-AS1 regulates the autophagy pathway and autophagic degradation of fusion proteins by inducing the expression of LAMP5, which was revealed to suppress autophagy [29, 31]. Disrupting the LAMP5-AS1-DOT1L-LAMP5 axis enhances the autophagic degradation of MLL fusion proteins, impairing the maintenance of MLL fusion protein. These findings highlight the potential of targeting lncRNAs that fine-tune autophagy to mediate the degradation of fusion proteins in aggressive cancers.

## Conclusions

The mechanism described here highlights the role of LAMP5-AS1 in *MLL* leukemia progression via regulating the autophagy pathway and autophagic degradation of MLL fusion proteins. Notably, the interaction of LAMP5-AS1 with DOT1L regulates H3K79 methylation directly at *LAMP5* locus, which sheds light on the complexity and underlying mechanism of *MLL* leukemia. It would be interesting to further explore the role of other LAMP5-AS1-like lncRNAs in the degradation of fusion proteins of transcription factors in aggressive cancers.

### Electronic supplementary material

Below is the link to the electronic supplementary material.

Supplementary Material 1

## Data Availability

The materials and data supporting the conclusion of this study have been included within the article.

## Abbreviations

lncRNA: Long noncoding RNA
DOT1L: DOT1 Like Histone Lysine Methyltransferase
LAMP5: Lysosome-associated membrane protein 5
LAMP5-AS1: LAMP5 antisense 1
MLL: Mixed-lineage leukemia
H3K79: Histone 3 lysine 79
LC3: MAP1LC3, microtubule-associated protein 1 light chain 3
qRT-PCR: Quantitative reverse transcription-PCR
siRNAs: Small interfering RNAs
shRNA: Short hairpin RNA
ChIP: Chromatin immunoprecipitation
FISH: RNA and DNA fluorescence in situ hybridizatioin
TEM: Transmission electron microscopy
ChIRP: Chromatin isolation by RNA purification
FBS: Fetal bovine serum
IF: Immunofluorescence
NOD/SCID: Nonobese diabetic/Severe combined immunodeficient
RFP: Red Fluorescent Protein
GFP: Green Fluorescent protein
GEO: Gene expression omnibus
