# Supplementary material for: Blockade of the lncRNA-DOT1L-LAMP5 axis enhances autophagy and promotes degradation of MLL fusion proteins
Source: Exp Hematol Oncol. 2024 Feb 19;13:18. doi: 10.1186/s40164-024-00488-5 (PMC10877858; doi:10.1186/s40164-024-00488-5)
Supplement: Supplementary file 1 — Supplementary Material 1 [file 40164_2024_488_MOESM1_ESM.docx]

Supplementary Materials


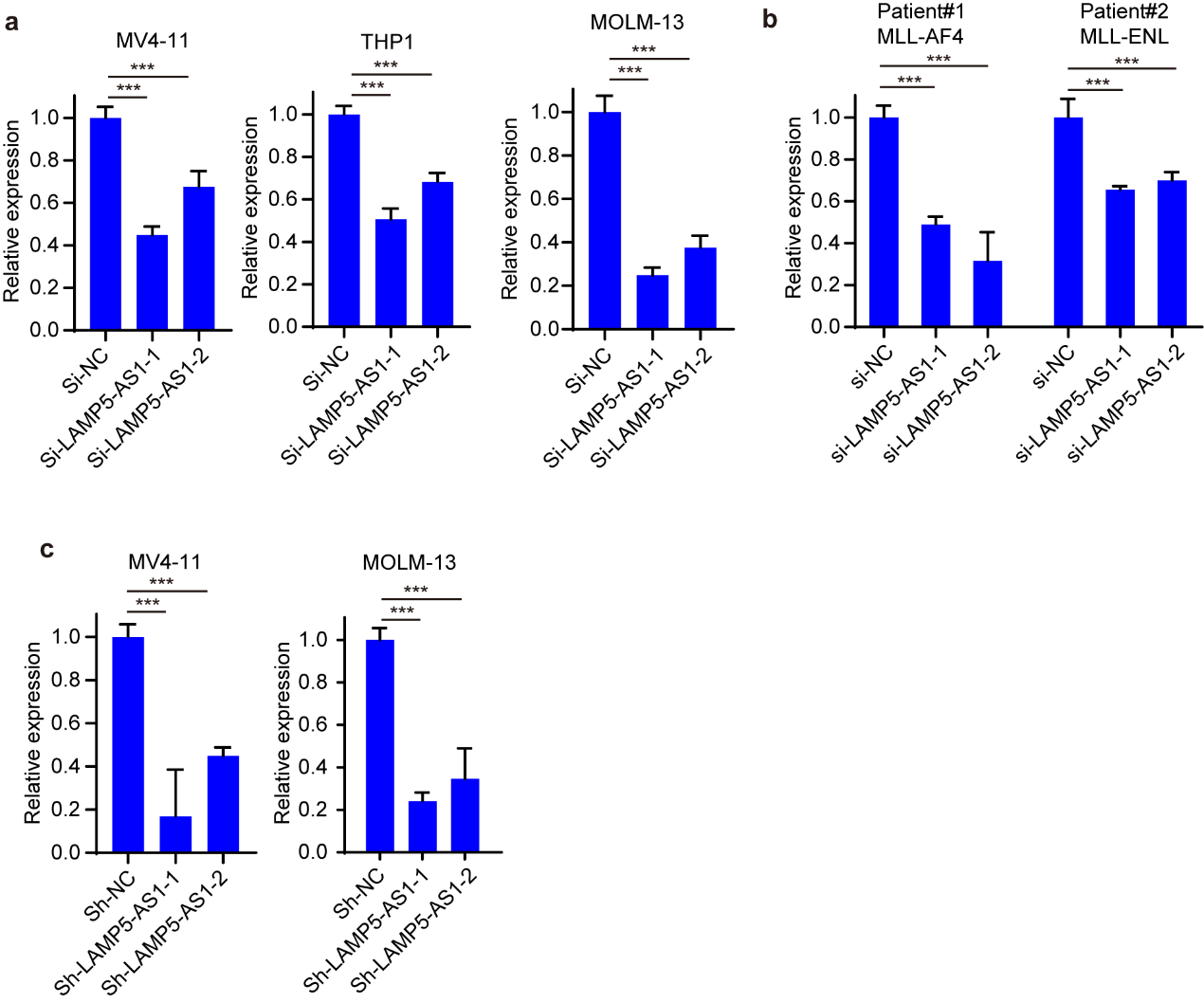


**Supplementary** **Fig. 1 LAMP5-AS1 is knocked down significantly by siRNA or shRNAs.** **a, b** Quantitative RT-PCR showing the knockdown of LAMP5-AS1 by siRNAs in *MLL* leukemia cell lines(**a**) and primary cells(**b**). **c** The lentivirus-infection efficiency of stable downregulation of LMAP5-AS1 by shRNAs in MV4-11 and MOLM-13. Error bars reflect ± SEM (***, p<0.001) from three independent experiments.


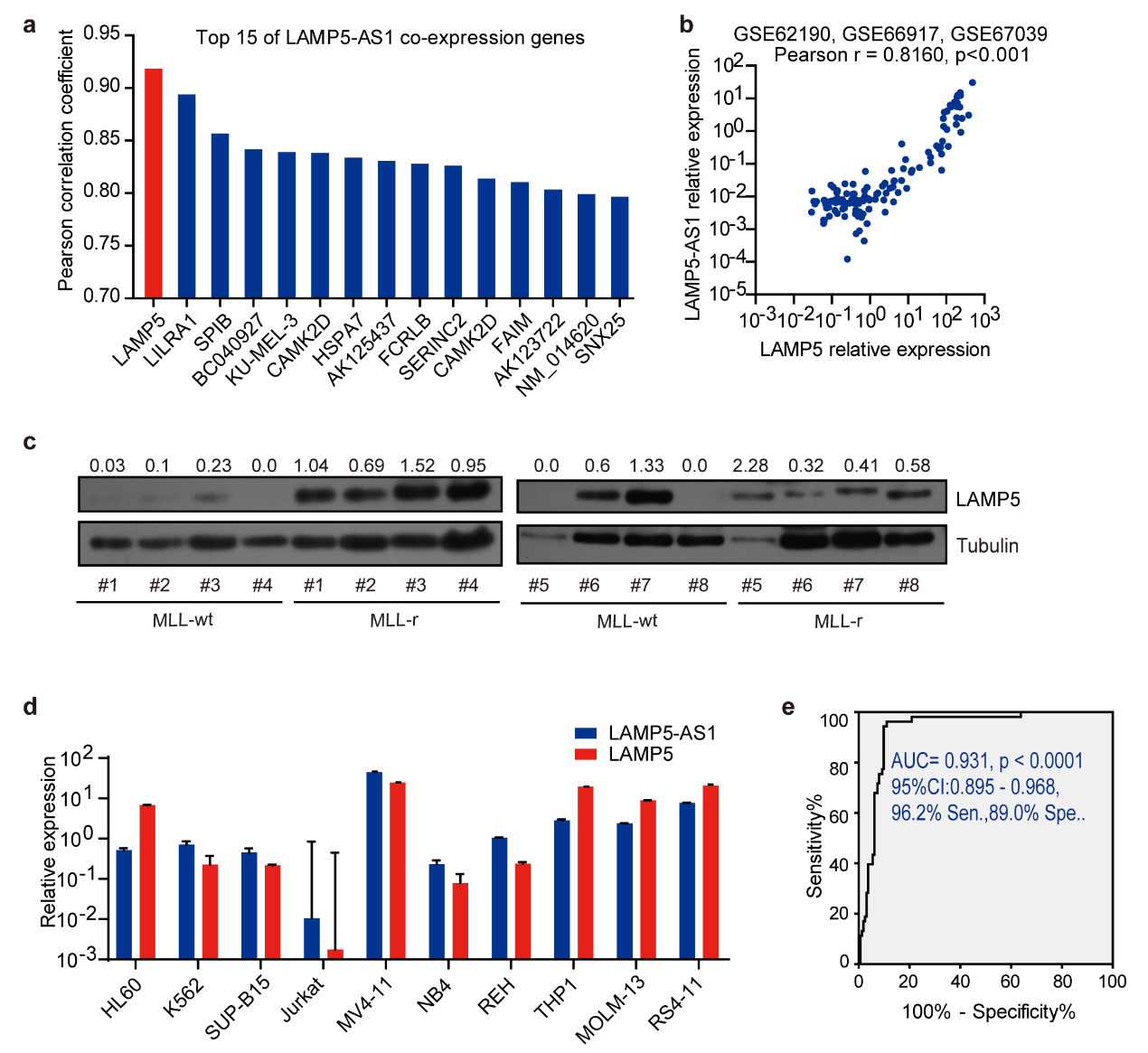


**Supplementary** **Fig. 2 The positive correction of LAMP5-AS1 and LAMP5 expression in leukemia**. **a** By analyzing our RNA-seq data in MLL and MLL-wt leukemia patient samples, the co-expression pattern between mRNA and lncRNA showed that the lysosome-associated membrane protein LAMP5, which is adjacent to LAMP5-AS1, is the most relevant with its antisense LAMP5-AS1. **b** The co-expression of LAMP5-AS1 and LAMP5 in leukemia patient samples (GSE62190, GSE66917, GSE67039). Pearson method was used to analyze the correlation coefficient. **c** Western blot for LAMP5 expression in *MLL*-wt and *MLL*-rearranged (*MLL*-r) leukemia patient samples. Tubulin regards as the negative control. The LAMP5 /Tubulin densitometric ratio were recorded by ImageJ. **d** The expression levels of LAMP5-AS1 and LAMP5 in the leukemia cell lines. **e** The ROC analysis for combination of LAMP5-AS1 and LAMP5 expression to discriminate the *MLL-*r or *MLL*-wt leukemia sets.


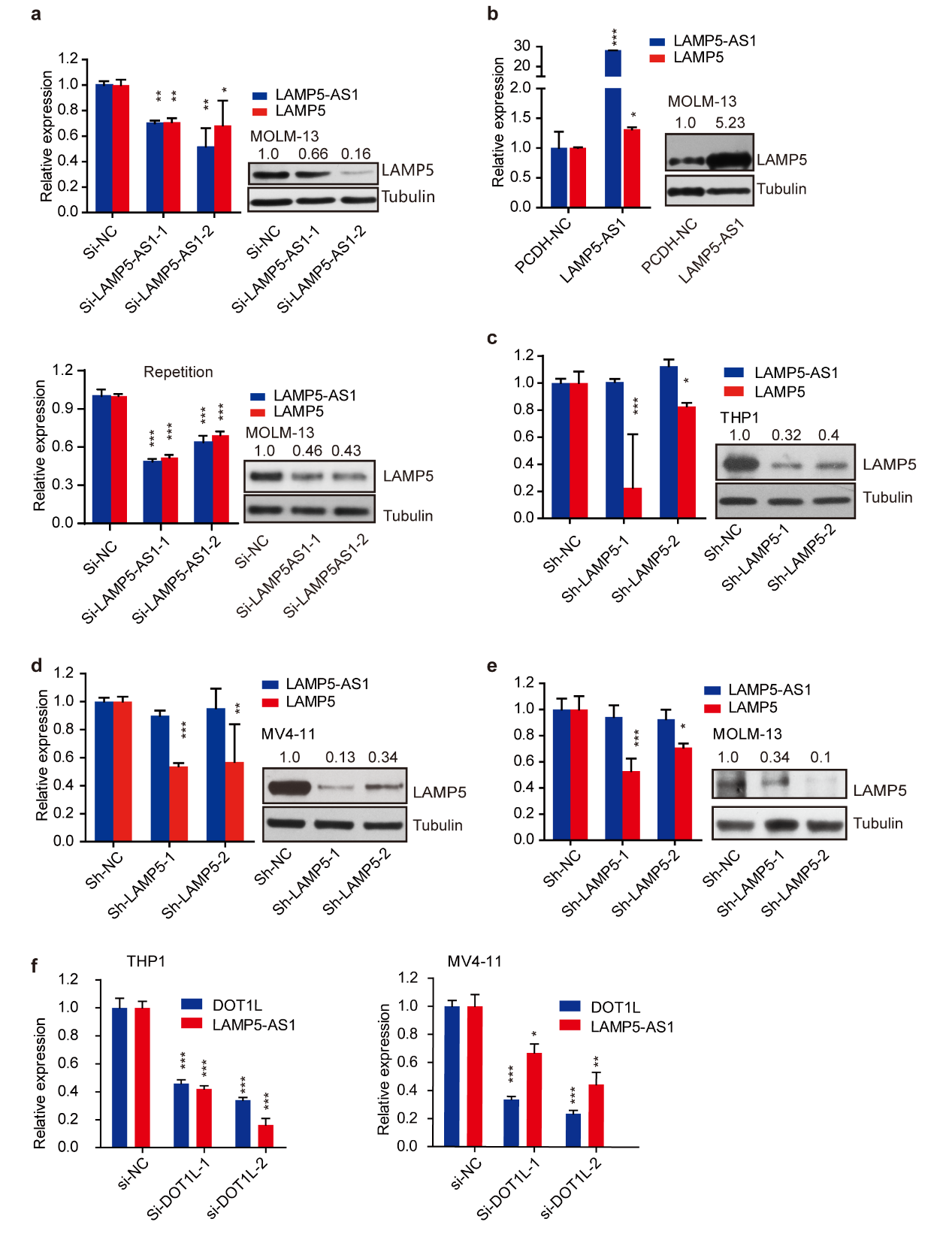


**Supplementary** **Fig. 3 LAMP5 is the downstream target of LMAP5-AS1. a** Quantitative RT-PCR and western blotting show that knocking down LAMP5-AS1 by siRNAs reduce LAMP5 expression in MOLM-13. **b** overexpression of LAMP5-AS1 increased LAMP5 expression in *MLL* leukemia cells. **c-e** Quantitative RT-PCR and western blotting the expression of LAMP5-AS1 and LAMP5 under Knocking down LAMP5 by siRNAs. **f** Quantitative RT-PCR and western blotting the expression of LAMP5-AS1 and DOT1L under Knocking down DOT1L by siRNAs. Error bars reflect ± SEM (*, p<0.05; **, p<0.01; **, p<0.001) from three independent experiments. The LAMP5 /Tubulin densitometric ratio were recorded by ImageJ.


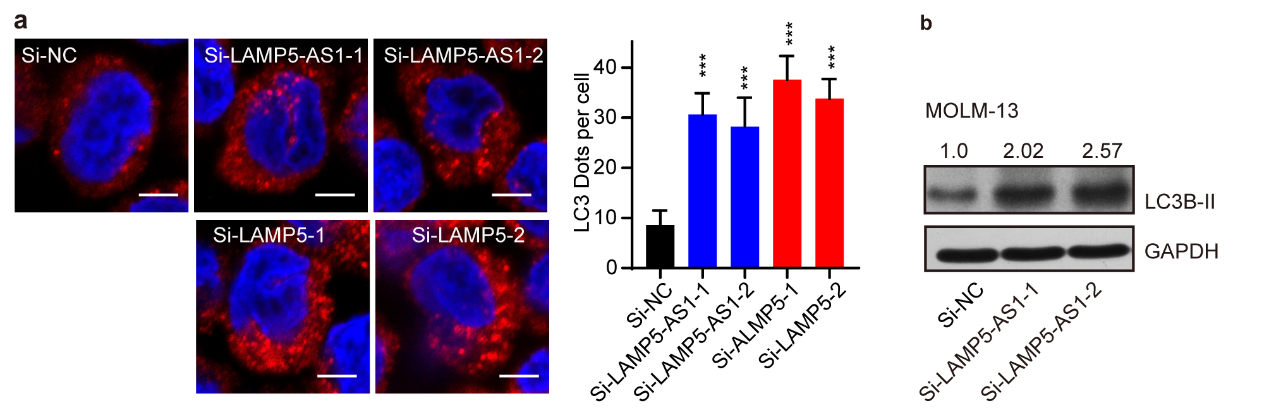


**Supplementary** **Fig. 4 LAMP5-AS1 regulates cell autophagy. a** Representative IF graphs (left) showing LC3B puncta accumulation in MOLM-13 cells. Histogram plots show the statistical values for the number of LC3B puncta per cell calculated by Image-Pro Plus (right, n = 20 cells; Error bars reflect ± SEM, ***, P <0.001). Scale bar, 10 mm. **b** Western blot showing LC3B-II enrichment after LAMP5-AS1 knockdown in MOLM-13 cells. The LC3B-II /Tubulin densitometric ratio were recorded by ImageJ.


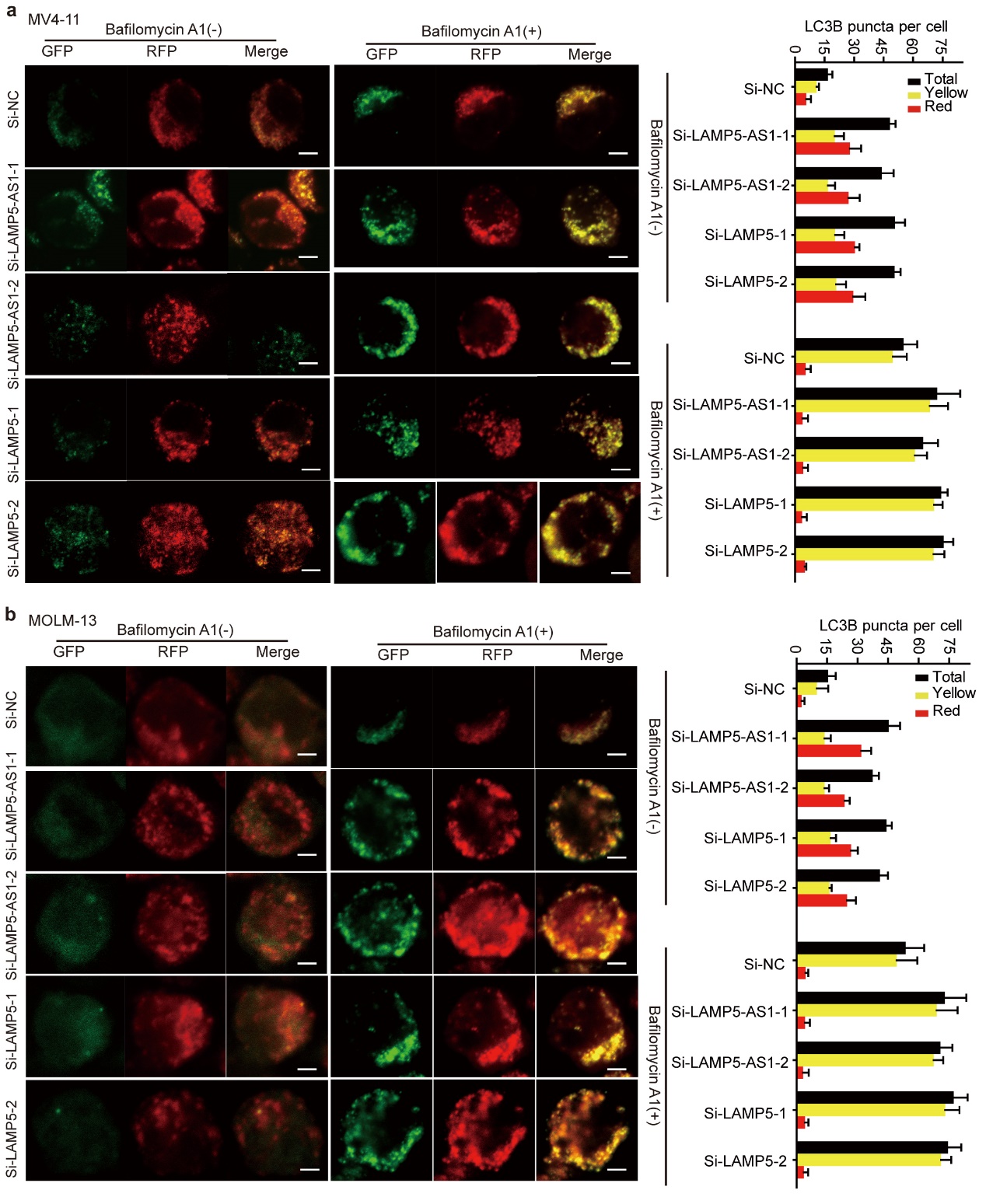


**Supplementary Fig. 5 LAMP5-AS1-LAMP5 axis regulates autophagic flux. a, b** siRNAs targeting LAMP5-AS1 and LAMP5 were transient transfected into stable mRFP-GFP-LC3 MV4-11(**a**) and MOLM-13 (**b**) cells which were treated with or without bafilomycin A1 (25 nM, 12 h). Scale bar, 10 μm. The numbers of yellow LC3 puncta and red LC3 puncta per cell in each condition were quantified using Image-Pro Plus. Total LC3 puncta represent the number of yellow LC3 puncta and red LC3 puncta. More than 20 cells were counted in each condition (Mean ± SEM).

**Supplementary Table S1. Clinicopathologic features of primary *MLL* leukemia patient samples.**

| Category | Age at diagnosis(years) | WBC count, × 10^9^/L | Fusion gene | Sex | AML/ALL |
| --- | --- | --- | --- | --- | --- |
| Patient 1 | 0.58 | 645.84 | *MLL-AF4* | Male | B-ALL |
| Patient 2 | 0.17 | 119.9 | *MLL-ENL* | Female | B-ALL |
| Patient 3 | 2 | / | *MLL-AF10* | Male | AML |
| Patient 4 | 5.75 | 20.5 | *MLL-AF9* | Male | B-ALL |

**Supplementary Table S2. The primers used in this work.**

| primers | | sequences（5' → 3'） | |  |
| --- | --- | --- | --- | --- |
| qPCR primers | | |  | |
| qLAMP5-AS1-Forward | CACTGAACGGATCTCAAACC | | | |
| qLAMP5-AS1-Reverse | CCAAGGGACAGTGATGCTAC | | | |
| qLAMP5-Forward | TCTAGTGATCCGCAGAAGACGG | | | |
| qLAMP5-Reverse | CAGGGGCAAGGTTTCTTCCAG | | | |
| qDOT1L-Forward | AAGAAGATGAACACTGCGAACC | | | |
| qDOT1L-Reverse | CTGGTAGAACGGGCTGTGAG | | | |
| qGAPDH-Forward | GAGTCAACGGATTTGGTCGTAT | | | |
| qGAPDH-Reverse | ATGGGTGGAATCATATTGGAAC | | | |
| ChIP primers for qPCR |  | | | |
| ChIP-LAMP5- Forward | CTGAGCCCTGAACTGATGGA | | | |
| ChIP-LAMP5- Reverse | CATTTTCCCGCACCACAA | | | |
| PCR primers |  | | | |
| LAMP5-AS1-1-Forward | CGGAATTCAAGCTAATTGACTTTACTTTGCC | | | |
| LAMP5-AS1-Reverse | CGGGATCCGACCTCATAAACTAATATTTATTGAATT | | | |
| LAMP5-AS1-AS-Forwad | TAATACGACTCACTATAGGGGACCTCATAAACTAATATTTATTGAATT | | | |
| LAMP5-AS1-AS-Reverse | aagctaattgactttactttgccggc | | | |
| SiRNA/shRNA |  | | | |
| Si-LAMP5-1-S | 5' GGCUGAGCAAAGUGCAGUU dTdT 3' | | | |
| Si-LAMP5-1-AS | 3' dTdT CCGACUCGUUUCACGUCAA 5' | | | |
| Si-LAMP5-2-S | 5‘ CAUACAAUGGCUCAAAUCA dTdT 3‘ | | | |
| Si-LAMP5-2-AS | 3‘ dTdT GUAUGUUACCGAGUUUAGU 5‘ | | | |
| Si-LAMP5-AS1-1-S | 5‘ CUGACAAAGUGCCGUCCAA dTdT 3‘ | | | |
| Si-LAMP5-AS1-1-AS | 3‘ dTdT GACUGUUUCACGGCAGGUU 5‘ | | | |
| Si-LAMP5-AS1-2-S | 5‘ GAGGCAAGACGAAGAAAGU dTdT 3 | | | |
| Si-LAMP5-AS1-2-AS | 3‘ dTdT CUCCGUUCUGCUUCUUUCA 5‘ | | | |
| Si-DOT1L-1-S | 5‘ GGAUGAAAUGGUAUGGAAA dTdT 3‘ | | | |
| Si-DOT1L-1-AS | 3‘ dTdT CCUACUUUACCAUACCUUU 5‘ | | | |
| Si-DOT1L-2-S | 5‘GUGCUCGAAUUGAGAGAAA dTdT 3‘ | | | |
| Si-DOT1L-2-AS | 3‘dTdT CACGAGCUUAACUCUCUUU 5‘ | | | |
| Sh-LAMP5-AS1-1-S | 5’GATCCCTGACAAAGTGCCGTCCAATTCAAGAGATTGGACGGCACTTTGTCAGTTTTTG3’ | | | |
| Sh-LAMP5-AS1-1-AS | 5’AATTCAAAAACTGACAAAGTGCCGTCCAATCTCTTGAATTGGACGGCACTTTGTCAGG3’ | | | |
| Sh-LAMP5-AS1-2-S | 5’GATCCGAGGCAAGACGAAGAAAGTTTCAAGAGAACTTTCTTCGTCTTGCCACTTTTTG3’ | | | |
| Sh-LAMP5-AS1-2-AS | 5’AATTCAAAAAGAGGCAAGACGAAGAAAGTTCTCTTGAAACTTTCTTCGTCTTGCCTCG3’ | | | |
| LAMP5-AS1 probe for ChIRP |  | | | |
| probe 1 | gcactctttgcagagaacag | | | |
| probe 2 | tgcgagtatggatctccaag | | | |
| probe 3 | aaggtaacaccacagaccac | | | |
| probe 4 | acagtagaatgcatcttcct | | | |
| probe 5 | tgggtacaaacttccgtaga | | | |
| probe 6 | gttgggaaatgagacgatcc | | | |
| probe 7 | gggtgtcaggacagggacaa | | | |
| probe 8 | ttggagcggagttggagttc | | | |
| probe 9 | tgggagaacgtattctgctg | | | |
| probe 10 | caggttctgtaaattgggca | | | |
| probe 11 | actcaggagtgcataagagc | | | |
| probe 12 | agtgggtatttctacactag | | | |
| probe 13 | tcaggctacagaatgtcagg | | | |
